# Editorial: Transplant Rejection and Tolerance—Advancing the Field through Integration of Computational and Experimental Investigation

**DOI:** 10.3389/fimmu.2017.00616

**Published:** 2017-05-30

**Authors:** Giorgio Raimondi, Kathryn J. Wood, Alan S. Perelson, Julia C. Arciero

**Affiliations:** ^1^Vascularized and Composite Allotransplantation Laboratory, Department of Plastic and Reconstructive Surgery, Johns Hopkins School of Medicine, Baltimore, MD, USA; ^2^Nuffield Department of Surgical Sciences, Oxford University, John Radcliffe Hospital, Oxford, UK; ^3^Theoretical Biology and Biophysics Group, Los Alamos National Laboratory, Los Alamos, NM, USA; ^4^Department of Mathematical Sciences, Indiana University – Purdue University Indianapolis, Indianapolis, IN, USA

**Keywords:** transplant rejection, transplant tolerance, theoretical modeling, big data and bioinformatics, mechanistic models, transplant immunology, biomarkers, systems biology

**The Editorial on the Research Topic**

**Transplant Rejection and Tolerance—Advancing the Field through Integration of Computational and Experimental Investigation**

Seventy years after the first proof of concept that the immune system can be trained to accept transplanted tissues *via* induction of immune tolerance, we are still waiting for a clinical approach that could be used routinely in transplant patients. Transplantation is a life-saving surgical procedure that is still only successful when paired with life-long administration of immunosuppressive drugs. However, the debilitating side effects of the long-term use of these drugs, together with their incomplete control of the immune system, compromise the quality of life and survival of transplant recipients. Thus, there is a strong push to find new therapeutic strategies that promote indefinite acceptance of a transplanted tissue without compromising the effectiveness of the patient’s immune system. Although many exciting ideas have been explored, none of the resulting strategies have been successfully converted into a widely applicable therapeutic approach.

Our knowledge of the complex immunological processes leading to transplant rejection continues to grow, and our understanding of the limitations associated with experimental models deepens. There is a great opportunity to foster a different approach to identify novel interventions. New tools of genomics, proteomics, and metabolomics are being implemented in powerful analyses that promise the development of better and safer personalized treatments. In parallel, theoretical modeling is slowly but progressively being welcomed among experimentalists due to its ability to unravel relevant mechanisms of complex systems and generate new hypotheses (1). The successful employment of these promising tools requires effective communication and collaboration among immunologists, data-driven modelers, and system biologists.

This Research Topic provides a venue for stimulating these interdisciplinary conversations in the context of transplantation. The articles collected under this Research Topic introduce new theoretical and experimental studies that describe novel techniques and methods for understanding the interactions between the immune response and transplants and for establishing more effective strategies of diagnosis and intervention that will promote transplant tolerance. The contributions of this Research Topic can be divided into two main groups according to the approaches they implement: (i) big data and bioinformatics and (ii) mechanistic and equation-based models of rejection.

To identify correlations and sensitivities from large data sets, various statistical methods and bioinformatics approaches are needed. Wang and Sarwal offer a concise review of the current uses and advances in statistical approaches and high-dimensional data applications for identifying possible transplant biomarkers. Identifying markers of injury, causative markers, and predictive markers is key for monitoring, managing patients, and identifying the re-purposing potential of existing drugs. Mastoridis et al. review current techniques (transcriptomic technologies) and propose future ideas for identifying biomarkers predictive of tolerance in the context of liver transplantation. They also explore how this knowledge could offer great insight into studying tolerance to other organs. In their perspective article, Stegall and Borrows argue that more accurate and mechanistic mathematical models can be designed to predict (renal) allograft loss or chronic injury, but they note that this will require access to more detailed molecular, histologic, and serologic data. Mechanistic studies conducted in parallel to focused clinical trials also would be tremendously useful for understanding why grafts fail and for designing tailored intervention.

Several statistical methods are applied to transplant data in articles of this collection to identify key biomarkers. Pike et al. used principle component analysis and other tools to analyze a large set of T cell immunophenotyping data before and after renal transplantation. They discovered that pretransplant frequency of programmed death 1 (PD-1) expressing T cell subsets stratifies patients at risk of developing rejection episodes. In a study of kidney transplants, Kadota et al. used various statistical algorithms to analyze the transcriptome of allograft biopsies and showed that histological classification of T cell mediated rejection contains multiple subtypes of rejection amenable to more personalized treatments. When studying the inflammatory response associated with ischemic injury, Starzl et al. combined principal component analysis and a regression approach to discover a cytokine-based signature to define the type and severity of the inflammatory response.

In transplant modeling, identifying the key players and interactions between transplants and the immune system is critical to understanding the pathway to rejection or tolerance. An agent-based model presented by An provides a dynamic and mechanistic understanding of transplant immunology so that control strategies to induce tolerance can be built. Arciero et al. provide one of the first comprehensive mathematical models of mouse heart transplant rejection. This ordinary differential equation-based model tracks innate and adaptive immunity and provides important suggestions of new investigations to improve the understanding of rejection. Day et al. present an ordinary differential equation model focused on the inflammatory response to surgical and ischemia/reperfusion injury. The model predicts specific conditions that lead to tolerance and others that lead to an exaggerated rejection response. Best et al. use a computational model of T cell repertoire development to examine self/non-self discrimination when incorporating features of cross-reactivity and T cell cooperativity. The resulting dynamic state of tolerance suggests specific opportunities for therapeutic intervention to achieve long-term tolerance.

Overall, all of the contributions to this Research Topic highlight the still largely untapped potential of integrating data-driven and mechanistic modeling into the “ordinary” experimental scientific approach to address key questions of transplant immunology in academic settings. As noted at a recent workshop of computational and experimental immunologists convened by the NIAID (2), there is still a broad divergence among researchers on how to approach fundamental immunological questions. This separation between modelers and experimentalists is even deeper in transplant immunology. However, all researchers share the common goal of improving the life of transplanted patients by understanding how to predict the behavior of immunological responses underlying graft rejection and failure. Despite the continuous growth of technological advances, it is still difficult to predict how a certain molecular or cellular intervention will affect the behavior of the entire system over time. This could be achieved, however, by properly integrating experimentation, data-driven modeling, and mechanistic modeling to test non-intuitive conditions impractical to explore using experimentation alone. The close collaboration between experimentalists and modelers necessary to reach this result requires a novel component of formal training of each part that will lead to productive communication and work integration. This Research Topic encourages the research community to embrace and implement this approach and witness exciting new discoveries that will ultimately benefit the patient population.

## Author Contributions

GR and JA prepared the first draft. KW and AP made critical revisions and provided valuable feedback.

## Conflict of Interest Statement

The authors declare that the research was conducted in the absence of any commercial or financial relationships that could be construed as a potential conflict of interest.
